# Supplementary material for: Drug Exposure and Susceptibility of Pyrazinamide Correlate with Treatment Response in Pyrazinamide-Susceptible Patients with Multidrug-Resistant Tuberculosis
Source: Pharmaceutics. 2024 Jan 21;16(1):144. doi: 10.3390/pharmaceutics16010144 (PMC10820138; doi:10.3390/pharmaceutics16010144)
Supplement: Supplementary file 1 [file pharmaceutics-16-00144-s001.zip › pharmaceutics-2612808-supplementary.pdf]

Supplementary Materials

Table S1. Univariable analysis results for risk factors of Pyrazinamide exposure, susceptibility, and treatment response

Characteristics	Pyrazinamide exposure and susceptibility			Treatment response			
	MIC OR(95%CI)	AUC Coefficient(95%CI)	AUC/MIC Coefficient(95%CI)	2-month culture conversion RR(95%CI)	6-month culture conversion RR(95%CI)	Treatment outcome RR(95%CI)	Time to culture conversion HR(95%CI)
Area							
Guizhou	1	1	1	1	1	1	1
Henan	0.92(0.22-3.73)	51.19(0.24-137.22)	1.35(-0.83-3.55)	1.64(0.93-2.90)	1.51(0.98-2.34)	1.19(0.88-1.6)	1.43(1.04-1.97)
Jiangsu	1.24(0.19-8.09)	77.49(0.07-163.52)	2.22(0.02-4.41)*	1.21(0.65-2.25)	1.61(1.05-2.47)*	1.12(0.82-1.52)	1.34(0.80-2.23)
Female	1.46(0.35-5.92)	93.06(0-162.18)*	1.87(0.09-3.66)*	1.04(0.67-1.61)	1.07(0.80-1.44)	1.06(0.84-1.33)	1.07(0.25-4.48)
Age	0.96(0.23-3.92)	-4.64(0--1.35)*	-0.06(-0.15-0.01)	1(0.98-1.02)	1(0.98-1.01)	1.00(0.99-1.01)	1.00(0.45-2.23)
Height	0(0-0.01)*	-717.93(0--293.73)*	-7.68(-18.88-3.51)	5.89(0.28-123.36)	1.44(0.23-9.07)	1.55(0.40-6.05)	4.49(2.55-7.93)
Weight	0.96(0.23-3.91)*	-4.41(0--1.53)*	-0.05(-0.12-0.02)	1.00(0.98-1.02)	1(0.99-1.01)	1.00(0.99-1.01)	1.01(0.68-1.50)
BMI	0.88(0.21-3.6)	-16.15(0.01--3.50)*	-0.23(-0.55-0.09)	1.00(0.92-1.09)	1(0.95-1.06)	1.02(0.98-1.06)	1.04(0.58-1.86)
Current smoker	1.07(0.26-4.36)	-61.23(0.08-8.51)	-1.06(-2.85-0.72)	0.68(0.44-1.06)	0.72(0.54-0.97)*	0.87(0.70-1.10)	0.77(0.51-1.16)
Diabetes mellitus type II	1.02(0.25-4.14)	-40.94(0.36-47.86)	-1.26(-3.53-1.00)	1.02(0.59-1.76)	0.84(0.55-1.29)	0.79(0.55-1.13)	0.72(0.44-1.17)
Pulmonary cavities	1.25(0.30-5.10)	16.85(0.64-88.7)	-0.54(-2.38-1.29)	0.94(0.6-1.49)	1.12(0.83-1.5)	0.9(0.70-1.15)	0.91(0.24-3.41)
Severe disease	1.98(0.48-8.05)	-47.59(0.26-36.64)	-2.96(-5.07--0.85)*	0.38(0.16-0.88)*	0.52(0.3-0.89)*	0.51(0.32-0.81)*	0.35(0.35-0.35)*
Time to positivity	0.83(0.20-3.39)*	0.72(0.89-11.24)	0.52(0.27-0.78)*	1.13(1.06-1.21)*	1.08(1.03-1.13)*	1.07(1.03-1.10)*	1.16(1.16-1.16)*
Pyrazinamide dose	1.06(0.26-4.33)	9.97(0-15.83)*	0.14(-0.01-0.29)	0.98(0.95-1.02)	0.99(0.97-1.02)	0.99(0.97-1.01)	0.98(0.45-2.11)
Group A drugs							
MFX+BDQ+LZD	1	1	1	1	1	1	1
MFX+LZD	1.77(0.43-7.22)	-55.85(0.20-29.62)	-3.32(-5.42--1.22)*	0.47(0.3-0.73)*	0.64(0.48-0.84)*	0.83(0.68-1.02)	0.47(0.47-0.47)*
MFX	3.81(0.58-24.83)*	-129.51(0--33.64)*	-5.5(-7.85--3.14)*	0.37(0.19-0.68)*	0.39(0.24-0.63)*	0.50(0.34-0.74)*	0.23(0.23-0.23)*
LZD	11.61(1.18-113.64)	55.19(0.52-223.53)	-4.69(-8.83--0.56)*	0.01(0.01-0.01)*	0.68(0.35-1.33)	0.66(0.34-1.28)	0.32(0.30-0.34)*

Table S2. Adverse events during the treatment in correspondence to the treatment categories.

**Adverse events* (n=132)**	**All patients (n=143) No.(%)**	**No.(%) of patients treated with**			
		**MFX+BDQ+LZD (n=37)**	**MFX+LZD (n=7)**	**MFX (n=64)**	**LZD (n=35)**
Gastrointestinal disorders	38(26.6)	11	2	16	9
Psychiatric disorders	18(12.6)	5	1	8	4
Central nerve system disorders	6(4.2)	2	1	3	2
Peripheral neuropathy	21(14.7)	6	2	3	5
Liver injury	10(7.0)	3	0	5	2
Kidney injury	4(2.8)	1	0	2	1
Anemia	11(7.7)	7	2	0	6
OTcF prolongation	16(11.2)	6	0	5	1
Others (pain, skin hyperpigmentation, et al)	8(5.6)	2	0	4	2

MFX: Moxifloxacin; BDQ: Bedaquiline; LZD: Linezolid;

*Adverse events were defined as follows:

Gastrointestinal disorders: the presence of nausea, vomiting, abdominal pain, diarrhea, epigastric discomfort, hematemesis, melena, and positive endoscopic findings;

Psychiatric disorders: the presence of depression, anxiety, psychosis, suicide, nightmares, and convulsion;

Central nervous system disorders: headache and seizure activity as reported by patient or witness;

Peripheral neuropathy: numbness, weakness, tingling, dizziness, burning/pain in the extremities, diagnosed by physician or electromyography;

Liver injury: alanine transaminase elevations above five times the upper limit of normal (ULN) or above three times the ULN with total bilirubin above 2 times the ULN;

Kidney injury: an abnormal estimated glomerular filtration rate based on serum creatinine using the Modification of Diet in Renal Disease formula;

Anaemia: hemoglobin level below 120 g/L for women and 130 g/L for men;

QTc prolongation: a corrected QT interval with the use of Fridericia's formula of more than 450 msec.
